# Spectrally resolved photon echo spectroscopy of Zn(II), Co(II) and Ni(II)–octaethyl porphyrins

**DOI:** 10.1016/j.cplett.2009.06.004

**Published:** 2009-07-07

**Authors:** S.K. Karthick Kumar, Vivek Tiwari, Tapas Goswami, Debabrata Goswami

**Affiliations:** a Department of Chemistry, Indian Institute of Technology, Kanpur 208016, India; b Center for Laser Technology, Indian Institute of Technology, Kanpur 208016, India

## Abstract

Spectrally resolved femtosecond three-pulse photon echo signal from some metal–octaethyl porphyrins (OEPs) like Zn(II)–OEP, Ni(II)–OEP, Co(II)–OEP is reported. Excited state dynamics is studied by time evolving photon echo spectra for different values of coherence and population relaxation times. Dependence on the spectrally resolved photon echo spectra on varying metal center is analyzed. For all these metallo-porphyrins, the electronic relaxation timescale is found to be limited by our laser pulsewidth of 50 fs whereas the timescale for intramolecular vibrational relaxation, occurring within the Q_00_ band was found to be over a picosecond for Co(II)–OEP and Ni(II)–OEP and within a picosecond for Zn(II)–OEP.

## Graphical abstract

Spectrally resolved femtosecond three-pulse photon echo signal from some metal–octaethyl porphyrins (M–OEPs) like Zn(II)–OEP, Ni(II)–OEP, Co(II)–OEP is reported.

## 1 Introduction

Developments in nonlinear spectroscopy and its various applications in time and frequency domain techniques have been giving valuable information about the dynamics of molecules at a variety of time scales. Use of multiple computer-interfaced delays for controlling coherence and population times provides population relaxation time, dephasing time, inhomogeneous broadening and vibrational structure of transient species. Coherent nonlinear spectroscopy using multiple femtosecond pulses is capable of providing detailed dynamical and spectroscopic information even in the presence of strong inhomogeneous broadening. Spectrally resolved one-color and two-color three-pulse photon echo (SRPE) spectroscopy in the visible region were used for probing vibrational and electronic dynamics of simple and complex molecular systems [1–5]. Temporally resolving the photon echo spectra for different excitation wavelengths gives detailed information about the induced nonlinear polarization. Book and Scherer used spectrally resolved stimulated photon echo to observe short term dynamics in a dye molecule that are not apparent in time gated or wavelength integrated photon echo techniques [6]. This technique was used to study exciton dynamics in GaAs and quantum wells [7,8]. These advancements in experimental techniques have accelerated the studies on the photophysical dynamics of metallo-porphyrins.

The porphyrin molecule which belongs to a class of cyclic tetrapyrrole compounds is an essential constituent of many biochemical pathways. Metallo-porphyrins play a major in two of the nature’s most important biological processes, the conversion of sunlight into energy in plants using chlorophyll and the transport of oxygen in blood using haemoglobin [9]. Also, metallo-porphyrins have potential in artificial solar energy harvesting systems and molecular logic gates [10,11]. Porphyrins, in general, have two sets of electronic transitions in the visible region: the Q band at 500–600 nm and the Soret band (B) at around 400–430 nm. All these bands are interpreted as (π–π^∗^) in origin with B representing strongly allowed excited state and the Q representing quasi allowed excited state [12]. In metallo-porphyrins, typically two visible bands are observed around 500–600 nm separated by ∼1250 cm^−1^. The lower energy band, Q_00_, is the electronic origin of the lowest-energy excited singlet state, while the higher energy band includes one mode of vibrational excitation denoted by Q_10_.

The Soret band absorption is intense because the Stokes shift associated with the porphyrin macrocycle is very small, hence it can reabsorb a significant fraction of the corresponding resonance fluorescence [13]. It is known that there is a general shift in the wavelength and intensity variation in the linear absorption spectrum due to external substituents on the ring. Any substitution at the metal center for a given set of external substituents also causes a wavelength shift [12]. According to Gouterman’s four orbital model, both the absorption bands arise from the linear combination of the nearly-degenerate electronic states within the quasi D_4h_ molecule and it accurately describes the effect of substituents on porphyrin spectra. The small metal perturbation, in case of regular porphyrins, on the absorption and emission spectra can be understood as a small perturbation of metal on the π-electrons of the porphyrin ring. In case of irregular porphyrins, the metal orbitals, through stronger mixing with the ring orbitals or through the introduction of new low energy optical transitions, have much stronger effects on absorption and emission. Effect of metals on pthalocyanines and the structure property relationship was explored by degenerate four-wave mixing [14]. Excited state dynamics of porphyrins using time resolved absorption techniques were extensively studied earlier using time resolved spectroscopy [15–22]. Large third-order nonlinear properties of core-modified expanded porphyrins and their structure–property correlations have been extensively studied in our group [23–25].

Due to short lifetimes of upper emitting states, most of the photophysical study of metallo-porphyrins has been focused on the lowest excited state and the lowest triplet state. In this Letter, we report on a multidimensional technique of spectrally resolved three-pulse photon echo signal from few metallo-porphyrins like Zn(II), Ni(II) and Co(II)–octaethyl porphyrins. Due to electronic degeneracy of the first excited state (Table 1), a femtosecond pulse excitation of either the Soret or the Q bands prepares a superposition of states whose dephasing and relaxation influences the time resolved signal. The first time delay, *t*_12_, between the first and second pulse and the second time delay, *t*_23_, between the second and third pulse is capable of revealing the coherence and population dynamics, respectively. The advantage of spectrally resolving the measured signal is that it provides a control of coherence time as an experimental variable. Hence, by varying the coherence time and measuring the corresponding spectrum, it is possible to study both the ground and excited state dynamics [3]. Our two-dimensional study of several metallo-porphyrins thus explores the evolution of the ground and excited states and their corresponding coherence behavior.

## 2 Theoretical background

**Fig. 1:**
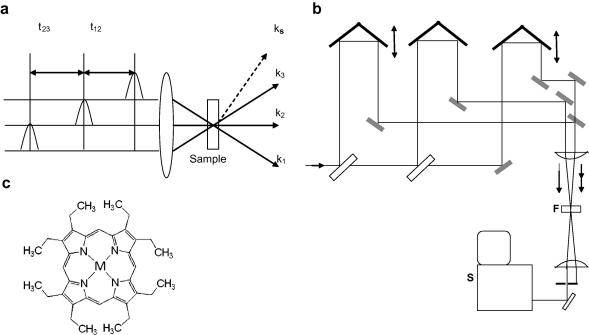
(a) Schematic diagram of the photon echo experiment. (b) Schematic of the experimental setup: S = spectrometer, F = flow cell. (c) General structure of metallo-porphyrins used. In our experiments, M = Co(II), Ni(II), Zn(II).

Photon echo experiments are based on a non-collinear four-wave mixing scheme with a specific phase matching function, where the incident beams propagate slightly differently in space (Fig. 1). Here three pulses are used with wavevectors *k*_1_, *k*_2_ and a probe pulse *k*_3_. The first pulse coherently excited the molecule at optical transition and second pulse *k*_2_ interacts with the freely evolving system giving rise to population grating in the space matching direction, whose spacing depends on the time interval between the first and second pulse, i.e. coherence time *t*_12_. A third pulse *k*_3_ separated from second pulse by a time *t*_23_ gets scattered due to the population grating and an echo signal is emitted in the direction, *k*_s_ = −*k*_1_ + *k*_2_ + *k*_3_. The non-collinear phase matching method has the advantage that signal is spatially separated from the incoming fields.

In four-wave mixing spectroscopy, external field *E*(*t*) is given by

$$E(t)=E_{1}(t+t_{12}+t_{23})e^{{\mathrm{ik}}_{1}\cdot r-i\omega_{1}t}+E_{2}(t+t_{23})e^{{\mathrm{ik}}_{2}\cdot r-i\omega_{2}t}+E_{3}(t)e^{{\mathrm{ik}}_{3}\cdot r-i\omega_{3}t}+c.c\text{.}$$

*E_j_*(*t*), *ω_j_* and *k_j_* denote time profile, mean frequency and wave vector of the *j*th incident field. The third-order nonlinear polarization *P*^(3)^ is related to the third-order response function *R*^(3)^ through the relation [26]

$$P^{\left(3\right)}(t\text{,}t_{12}\text{,}t_{23})=\int_{0}^{\infty }{\mathrm{dt}}_{3}\int_{0}^{\infty }{\mathrm{dt}}_{2}\int_{0}^{\infty }{\mathrm{dt}}_{1}E_{1}^{*}(t+t_{12}+t_{23}-t_{3}-t_{2}-t_{1})E_{2}(t+t_{23}-t_{3}-t_{2})E_{3}(t-t_{3})R^{\left(3\right)}(t_{3}\text{,}t_{2}\text{,}t_{1})$$

and the measured nonlinear signal is given by

$$S(t)∼|P^{\left(3\right)}(t){|}^{2}\text{.}$$

We consider here the signal from the direction $k_{s}=k_{3}+k_{2}-k_{1}$ and $\omega_{s}=\omega_{1}=\omega_{2}=\omega_{3}$. To obtain information about the temporal evolution of nonlinear polarization, the spectrum of the echo signal can be recorded. Then the frequency domain third-order nonlinear polarization is obtained by Fourier transformation of *P*^(3)^ with respect to *t*, i.e.

$$P^{\left(3\right)}(\omega \text{,}t_{12}\text{,}t_{23})=\int_{-\infty }^{+\infty }P^{\left(3\right)}(t\text{,}t_{12}\text{,}t_{23})e^{i\omega t}\mathrm{dt}\text{.}$$

The time integrated frequency domain signal field radiated by the polarization *P*^(3)^ is given by

$$E_{s}(\omega \text{,}t_{12}\text{,}t_{23})\propto [2\pi \mathrm{iln}(\omega )c]P^{\left(3\right)}(\omega \text{,}t_{12}\text{,}t_{23})$$

where *n*(*ω*) is the real part of the refractive index of the sample at frequency *ω*, *l* is the length of the sample and *c* is the speed of light. The intensity of spectrally resolved photon echo signal is then given by [6]

$$S_{\text{SRPE}}(\lambda_{d}\text{,}t_{12}\text{,}t_{23})\propto |E_{s}(\omega \text{,}t_{12}\text{,}t_{23}){|}^{2}\propto |P^{\left(3\right)}(\lambda_{d}\text{,}t_{12}\text{,}t_{23}){|}^{2}\text{,}$$

where *λ_d_* is the detection wavelength that is used to amplify the echo signal emerging along the *k*_s_ direction (Fig. 1a).

## 3 Experimental setup

A commercial Ti:sapphire multipass amplifier (ODIN, Quantronix Corp.) was seeded by 20 fs pulses at a wavelength of 800 nm from a Ti:Sapphire oscillator (KM Labs.) and pumped by a Nd:YAG laser operating at 1 kHz (Corona, Coherent Inc.). This amplified laser generated 40 fs pulses with the central wavelength at 806 nm, which was used to pump a commercial computer controlled travelling wave optical parametric amplifier (TOPAS, LightConversion Ltd.). In the optical parametric amplifier (OPA), a small fraction of the pump from multipass amplifier was used to generate the parametric superfluorescence seed in a thick BBO crystal and was tunable across the visible region. Visible pulses of 50 fs pulse width from the OPA was split into three beams of equal intensity by using appropriate thin ultrafast beam splitters and sent through variable delay lines consisting of retroflectors and motorized translation stages (Fig. 1a and b). The pulses were in boxcar geometry, i.e., with pulses placed on three corners of a square of 1 cm length. The three pulses are finally attenuated and focused using an achromatic lens of 20 cm focal length on the sample of unit optical density at 545 nm, however, kept flowing in an ultrathin fused silica flow-cell of 200 m path-length to avoid propagation effects [27]. Teflon tubing was used in the flow cell setup and a peristaltic pump was used to circulate the sample, such that a fresh portion of sample interacted with the pulses every time. It was important to flow the sample because of the rapid photo-degradation of porphyrins dissolved in dichloromethane. Retroreflectors placed on translation stages (Newport, UTM-CC-0.1) were used to precisely control the time delays between the pulses. The generated photon echo signal in the direction *k*_s_ was dispersed in a monochromator (Acton-SP150) attached with a thermoelectric cooled charge coupled device (CCD) camera. For a given SRPE measurement, the t_12_ was set fixed and the spectrum was recorded for population time *t*_23_ varying from −400 fs to +400 fs. The metallo-porphyrin samples (Fig. 1c) of Zn(II), Ni(II) and Co(II)–octaethyl porphyrins (OEP) were purchased from Sigma–Aldrich and were used without further purification and dissolved in dichloromethane.

## 4 Results and discussion

**Fig. 2:**
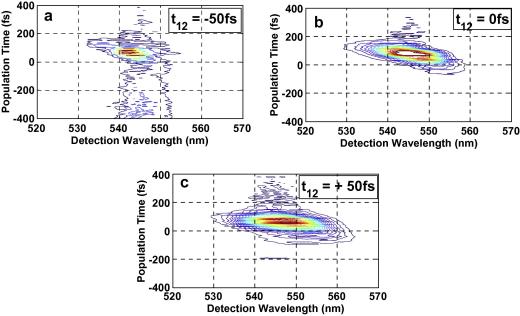
(a) One color three-pulse photon echo spectra (central wavelength 550 nm) of Zn–OEP in the direction *k*_s_ = − *k*_1_ + *k*_2_ + *k*_3_ versus population time *t*_23_ for *t*_12_ = −50 fs. (b) One colour three-pulse photon echo spectra (central wavelength 550 nm) of Zn–OEP in the direction *k*_s_ = −*k*_1_ + *k*_2_ + *k*_3_ versus population time *t*_23_ for *t*_12_ = 0 fs. (c) One color three-pulse photon echo spectra (central wavelength 550 nm) of Zn–OEP in the direction *k*_s_ = −*k*_1_ + *k*_2_ + *k*_3_ versus population time *t*_23_ for *t*_12_ = +50 fs.

**Fig. 3:**
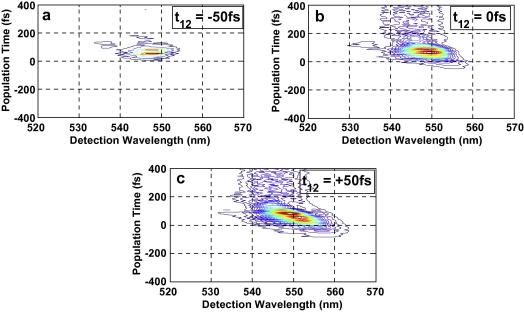
(a) One color three-pulse photon echo spectra (central wavelength 550 nm) of Ni–OEP in the direction *k*_s_ = − *k*_1_ + *k*_2_ + *k*_3_ versus population time *t*_23_ for *t*_12_ = −50 fs. (b) One color three-pulse photon echo spectra (central wavelength 550 nm) of Ni–OEP in the direction *k*_s_ = −*k*_1_ + *k*_2_ + *k*_3_ versus population time *t*_23_ for *t*_12_ = 0 fs. (c) One color three-pulse photon echo spectra (central wavelength 550 nm) of Ni–OEP in the direction *k*_s_ = −*k*_1_ + *k*_2_ + *k*_3_ versus population time *t*_23_ for *t*_12_ = +50 fs.

**Fig. 4:**
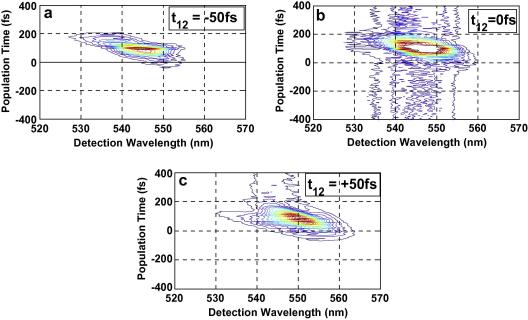
(a) One color three-pulse photon echo spectra (central wavelength 550 nm) of Co–OEP in the direction *k*_s_ = − *k*_1_ + *k*_2_ + *k*_3_ versus population time *t*_23_ for *t*_12_ = −50 fs. (b) One color three-pulse photon echo spectra (central wavelength 550 nm) of Co–OEP in the direction *k*_s_ = −*k*_1_ + *k*_2_ + *k*_3_ versus population time *t*_23_ for *t*_12_ = 0 fs. (c) One color three-pulse photon echo spectra (central wavelength 550 nm) of Co–OEP in the direction *k*_s_ = −*k*_1_ + *k*_2_ + *k*_3_ versus population time *t*_23_ for *t*_12_ = +50 fs.

The nonresonant excitation pulses were tuned to 545 nm central wavelength which is around Q_00_ for Co(II)–OEP and Ni(II)–OEP, and also overlapping with the longer wavelength part of the Q_10_ band. The spectra of excitation pulses also overlap with the longer wavelength of the Q_10_ band in case of Zn(II)–OEP. The spectrally resolved photon echo signal plots for the Zn(II)–OEP, Ni(II)–OEP and Co(II)–OEP in DCM, for varying population times is plotted in Figs. 2–4 for three coherence times *t*_12_ = −50 fs, 0 fs and +50 fs.

For positive population times, *t*_12_, the spectra shows a strong photon echo and population grating components. The strong photon echo component with the maximum intensity is observed at *t*_23_ within 100 fs. For negative coherence times, *t*_12_, the spectra exhibit weak signal. It can be observed from SRPE contour plots Figs. 2–4 that the peak of the spectrally resolved signal red-shifts from 545 nm to 550 nm as the coherence time is shifted from −50 fs to +50 fs. Significant coherence time dependent spectral broadening at early population times is observed in the photon echo spectra of all the three metal–OEPs. For positive coherence times the spectral broadening appears on the longer wavelength of the spectra. For negative coherence times the spectra are broadened on the shorter wavelength, while for coherence times near zero the spectra broadens relatively evenly on both the sides. The increasing red-shift with increasing population time *t*_23_ and more broadening for excitation wavelengths close to the absorption peak is reflective of the relaxation dynamics of the population [2,28,29]. A slight tilt can be observed in the SRPE contour plots for Ni(II)–OEP and Co(II)–OEP when the coherence time is positive which is indicative of inhomogeneous broadening as can be seen in Figs. 3c and 4c. Since we do not observe any tilt in the SRPE contour plot for Zn(II)–OEP, this suggests that inhomogeneous broadening occurs at a relatively longer timescale.

**Fig. 5:**
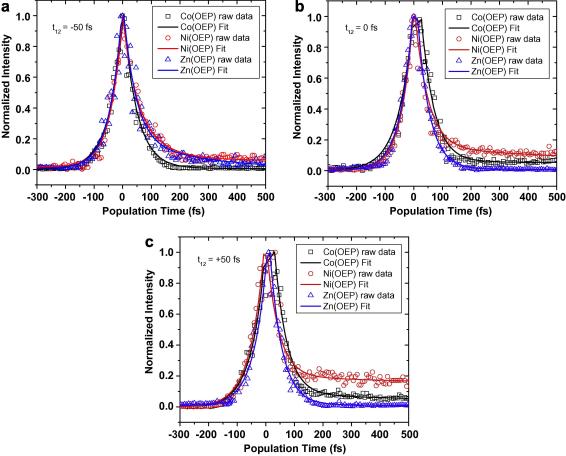
(a) Time-integrated three-pulse photon echo intensities for different samples at *t*_12_ = −50 fs. Shown in solid lines are the exponential rise and a bi-exponential decay fits for calculating the associated rise and decay timescales. (b) Time-integrated three-pulse photon echo intensities for different samples at *t*_12_ = 0 fs. Shown in solid lines are the exponential rise and a bi-exponential decay fits for calculating the associated rise and decay timescales. (c) Time-integrated three-pulse photon echo intensities for different samples at *t*_12_ = +50 fs. Shown in solid lines are the exponential rise and a bi-exponential decay fits for calculating the associated rise and decay timescales.

Our excitation pulse is in the long wavelength tail of the Q_01_ band. The time dependent signal is measured as function of delay of the third pulse with respect to the first two pulses that have a fixed delay between them (Fig. 1a). All the three pulses used in our experiment are sub-50 fs and as such, all the sub-50 fs timescales recovered from our experimental measurements is instrument resolution limited. We find the measured signals have a characteristic single exponential sub-50 fs rise followed by a decay that can be effectively fitted to a bi-exponential decay model. If the second pulse arrives 50 fs before the first pulse, the two decay timescales resulting from our bi-exponential decay model are 48 fs and 302 fs for Co(II)–OEP, 44 fs and 100 fs for Ni(II)–OEP and 43 fs and 280 fs for Zn(II)–OEP as shown in Fig. 5a. During zero coherence time, i.e., when the first and the second pulses arrive simultaneously, the two decay terms are respectively, 45 fs and 5 ps for Co(II)–OEP, 46 fs and 1.1 ps for Ni(II)–OEP and 42 fs and 100 fs for Zn(II)–OEP, as shown in Fig. 5b. Finally, when the coherence time is +50 fs, i.e., the second pulse arrives 50 fs after the first pulse, once again two decay terms are seen, which are 43 fs and 1.7 ps for Co(II)–OEP, 42 fs and 1 ps for Ni(II)–OEP and 42 fs and 192 fs for Zn(II)–OEP as shown in Fig. 5c. Thus, in all the cases, the fast component of the bi-exponential decay is also pulsewidth limited (sub-50 fs). The slow component is most prominent for Ni(II)–OEP and least prominent for Zn(II)–OEP. A comparison across the different cases in Fig. 5 shows that Zn-substituted porphyrins behave differently as compared to the Ni or Co substitution, which can perhaps be attributed to the filled d-shell of Zn. Furthermore, in case of Zn porphyrins, π–π interaction is more because of better planarity, as the Zn metal fits more easily into the porphyrin molecule. This can also be one of the reasons why its time constants are much faster than the other metallo-porphyrins. From these timescales we can infer that the ultrafast relaxation of the population placed on Q_10_ occurs in less than 50 fs and is pulsewidth limited in our case and is related to the Q_01_ to Q_00_ electronic relaxation. The longer relaxation timescale associated may be related to the intramolecular vibrational redistribution within the Q_00_ state.

## 5 Conclusion

We have presented spectrally resolved femtosecond three-pulse photon echo measurements on Zn(II)–OEP, Ni(II)–OEP and Co(II)–OEP. Increased degree of freedom in scans of time delays allows one to separate and extract specific type of spectroscopic information in complex molecules by studying spectral and temporal evolution of the photon echo signals. By varying the population times, population relaxation dynamics and inhomogeneous broadening is revealed in the photon echo spectra. Time-integrated photon echo signals show two different timescales. The electronic relaxation timescale is found to be sub 50 fs whereas the timescale for intramolecular vibrational relaxation, occurring in Q_00_ band, was found to be over a picosecond for Co(II)–OEP and Ni(II)–OEP and within a picosecond for Zn(II)–OEP.

## Figures and Tables

**Table 1:** Absorption peaks.

Band	Co(II)–OEP (DCM) (nm)	Ni(II)–OEP (DCM) (nm)	Zn(II)–OEP (DCM) (nm)
B(0, 0)	384	392	407
Q(1, 0)	520	516	536
Q(0, 0)	550	551	572
